# Morphometric Analysis of the Supraorbital Foramen and Notch in Adult North Indian Dry Skulls: Anatomical Variations With Clinical and Anthropological Implications

**DOI:** 10.7759/cureus.117469

**Published:** 2026-10-05

**Authors:** Gunjan Rai, Soumya Khanna, Prakash Kumar, Siddharth Verma, Ankita, Amrita Jyoti, Shweta Kumari

**Affiliations:** 1 Anatomy, Institute of Medical Sciences, Banaras Hindu University, Varanasi, IND; 2 Ophthalmology, Baba Kina Ram Autonomous State Medical College (ASMC) Chandauli, Chandauli, IND; 3 Anatomy, North Delhi Municipal Corporation (NDMC) Medical College and Hindu Rao Hospital, New Delhi, IND

**Keywords:** accessory supraorbital foramen, clinical anatomy, craniofacial anatomy, dry skull, forensic anthropology, morphometry, north indian population, regional anesthesia, supraorbital foramen, supraorbital notch

## Abstract

Background: The supraorbital foramen (SOF) and supraorbital notch (SON) are important anatomical landmarks transmitting the supraorbital neurovascular bundle. Their morphology and position exhibit considerable population-specific variation, directly influencing the safety and success of supraorbital nerve blocks, craniofacial and orbital surgeries, and reconstructive procedures. Despite their clinical and forensic significance, comprehensive morphometric data for the North Indian population remain limited.

Objective: This study aimed to evaluate the morphology, morphometric characteristics, and anatomical variations of the supraorbital foramen and notch in adult North Indian dry skulls and establish population-specific reference data with clinical and anthropological relevance.

Materials and methods: An observational study was conducted on 63 adult dry human skulls (126 supraorbital regions) obtained from the Department of Anatomy, Institute of Medical Sciences, Banaras Hindu University. Morphological variants of the supraorbital opening, presence and location of accessory foramina, foraminal shape, morphometric dimensions, and distances from established anatomical landmarks were recorded using a digital Vernier caliper. Measurements were performed in triplicate. Categorical variables were summarized as frequencies and percentages, and continuous variables as mean ± standard deviation. Independent-samples t-tests were used to compare right- and left-sided measurements of complete supraorbital foramina, whereas paired-samples t-tests were used for bilateral measurements obtained from the same skull. All tests were two-tailed, with p < 0.05 considered statistically significant.

Results: Supraorbital notch variants predominated, accounting for 76 (60.3%) of all supraorbital margins, while foramen variants constituted 32 (25.4%), combined notch-foramen variants constituted 14 (11.1%), and supraorbital depressions (SOD) constituted 4 (3.2%). Accessory supraorbital foramina were identified in 20 (15.9%) supraorbital regions, most commonly as a single lateral accessory foramen. Oval foramina represented the predominant shape, occurring in 19 (48.7%) of complete supraorbital foramina. The mean vertical and transverse diameters of complete foramina showed no significant bilateral differences. A statistically significant right-left difference was observed only for the distance from the nasion to the supraorbital opening (p < 0.001), indicating slight lateralization on the right side.

Conclusion: The examined North Indian osteological sample demonstrated a predominance of supraorbital notch variants, frequent accessory foramina, and measurable bilateral variation in selected anatomical relationships. These findings provide descriptive morphometric data that may be useful for comparative and clinical anatomical research.

## Introduction

The supraorbital margin of the frontal bone forms an important anatomical gateway through which the supraorbital neurovascular bundle exits the orbit to supply the forehead and anterior scalp [1]. Sensory innervation of this region is primarily provided by the supraorbital and supratrochlear nerves, the terminal branches of the frontal nerve arising from the ophthalmic division (V1) of the trigeminal nerve. The frontal nerve, a branch of the ophthalmic division of the trigeminal nerve, enters the orbit through the superior orbital fissure and courses anteriorly along the superior aspect of the levator palpebrae superioris muscle. Within the orbit, it divides into its two terminal branches, the supraorbital and supratrochlear nerves. The point of this division is variable in relation to the orbital length and may occur in either the proximal or distal half of the orbit [2]. The supraorbital nerve emerges through the supraorbital notch (SON) or supraorbital foramen (SOF), whereas the supratrochlear nerve exits medial to the supraorbital margin [1]. Owing to its superficial course and confinement at the superior orbital margin, the supraorbital neurovascular bundle is particularly susceptible to traumatic, compressive, and iatrogenic injury [3].

The morphology of the supraorbital opening demonstrates considerable anatomical variation. It may present as a complete foramen, an incomplete foramen, a notch, a notch associated with a foramen, or, less commonly, as a supraorbital depression (SOD). Variations also occur in its shape, size, position, and number [4].

These anatomical variations are of considerable clinical importance because they directly influence the course of the supraorbital nerve and its vulnerability during surgical and anesthetic procedures [1,2]. Accurate localization of the supraorbital nerve is essential for successful regional nerve blockade; however, variation in the position of the SOF/SON and the presence of accessory foramina may result in incomplete anesthesia when conventional anatomical landmarks are used [1,5]. Accessory foramina also increase the risk of neurovascular injury during brow-lift surgery, frontal craniotomy, orbital reconstruction, blepharoplasty, and cosmetic procedures involving the forehead [1,6,7]. Furthermore, narrow foramina or accessory osseous canals may predispose the supraorbital nerve to chronic compression, contributing to supraorbital neuralgia, persistent frontal headache, and migraine-like syndromes [8-11].

Numerous studies have demonstrated significant inter-population variation in the morphology and morphometry of the supraorbital notch and foramen, indicating that anatomical data derived from one population cannot be universally applied [12-18]. Population-specific morphometric information is therefore essential for improving the accuracy of regional anesthesia, craniofacial surgery, radiological interpretation, and forensic identification. However, comprehensive morphometric data describing the supraorbital region in the North Indian population remain limited, particularly with respect to accessory foramina and clinically relevant anatomical relationships.

In addition to its clinical importance, the supraorbital foramen and notch have anthropological and forensic significance because cranial morphometric traits exhibit population-specific variation and contribute to the assessment of ancestry and population affinity in skeletal remains [19]. Since most forensic reference standards are derived from Caucasian and East Asian populations, population-specific data from North Indian skulls provide valuable baseline information for anthropological research and forensic applications.

Therefore, the present study was undertaken to evaluate the morphology, morphometric characteristics, and anatomical variations of the supraorbital foramen and notch in adult North Indian dry skulls, with the aim of generating clinically relevant anatomical data to facilitate safer surgical, anesthetic, and diagnostic procedures involving the supraorbital region.

## Materials and methods

An observational morphometric study was conducted on 63 adult dry human skulls obtained from the Department of Anatomy, Institute of Medical Sciences, Banaras Hindu University (IMS-BHU), during 2022-2025. Institutional ethical approval was obtained from the Institutional Ethics Committee, IMS-BHU (Approval No. Dean/2022/EC/3619; dated October 20, 2022). The study was conducted in accordance with the principles of the Declaration of Helsinki. Skulls with intact frontal bones and supraorbital margins and clearly identifiable anatomical landmarks were included, whereas skulls showing fractures, gross pathology, or deformity involving the frontal region were excluded.

Each skull was positioned in the Frankfurt horizontal plane, and both supraorbital margins were examined, yielding 126 supraorbital regions for morphological assessment. The morphology of the supraorbital opening was classified as a supraorbital notch, complete supraorbital foramen, incomplete supraorbital foramen, or supraorbital depression according to previously described anatomical criteria [4,18]. A supraorbital notch was defined as an open indentation of the superior orbital margin without a complete osseous bridge, whereas a complete supraorbital foramen was defined as a completely enclosed osseous opening. An incomplete supraorbital foramen was defined as a partially ossified notch with an incomplete bony bridge. A supraorbital depression was recorded when a shallow concavity was present at the expected site of the supraorbital opening without formation of a distinct notch or complete foramen.

Landmark-based measurements were obtained from all 126 supraorbital sides, including supraorbital notches, complete and incomplete foramina, and supraorbital depressions. The center of the supraorbital opening was defined as the midpoint of the principal notch or foramen; in supraorbital depressions, the midpoint of the shallow concavity at the expected site of the supraorbital opening was used as the reference point.

The largest and most prominent opening on each side was designated as the main supraorbital opening, and any additional foramina were recorded as accessory supraorbital foramina. Their number and location were documented. Morphometric measurements included the maximum vertical and transverse diameters of complete supraorbital foramina, the distance from the nasion to the center of the supraorbital opening, the vertical distance from the inferior margin of the supraorbital foramen to the superior orbital rim, the distance from the center of the supraorbital opening to the frontozygomatic suture, and the distance from the center of the supraorbital opening to the temporal crest of the frontal bone. The center of the supraorbital opening was defined as the midpoint of the principal notch or foramen; in supraorbital depressions, the midpoint of the shallow concavity at the expected site of the supraorbital opening was used as the reference point. These measurements were based on previously published morphometric approaches [1,4,18].

Linear measurements were obtained using an Aerospace mechanical Vernier caliper (0-150 mm range; least count 0.01 mm). Each parameter was measured three times, and the mean value was recorded.

Data were entered into Microsoft Excel (Microsoft Corporation, Redmond, Washington) and analyzed using IBM SPSS Statistics for Windows, Version 25 (Released 2017; IBM Corp., Armonk, New York). Categorical variables were summarized using frequencies and percentages, whereas continuous variables were expressed as mean ± standard deviation (SD). The unit of analysis for morphological characteristics was the individual supraorbital region (right or left), with 126 regions assessed from 63 skulls.

For morphometric measurements of complete supraorbital foramina, right- and left-sided measurements were compared using an independent-samples t-test, as the right and left groups comprised separate observations of complete foramina (right, n = 21; left, n = 18). These analyses included vertical diameter, transverse diameter, and the vertical distance from the inferior margin of the supraorbital foramen to the superior orbital rim.

For measurements obtained from both sides of the same skull in relation to anatomical landmarks, right- and left-sided values were treated as paired observations, with each skull constituting one paired unit (63 right-left pairs). A paired-samples t-test was used to compare the distance from the nasion to the center of the supraorbital opening, the distance from the center of the supraorbital opening to the frontozygomatic suture, and the distance from the center of the supraorbital opening to the temporal crest of the frontal bone. All t-tests were two-tailed, and a p-value <0.05 was considered statistically significant.

## Results

A total of 63 adult dry human skulls (126 supraorbital regions) were examined. Morphological observations were recorded for all skulls, and landmark-based morphometric measurements were obtained. 

Morphological variations of the supraorbital opening

The distribution of morphological variants is presented in Table 1 and Figure 1. The isolated supraorbital notch was the most frequent variant, occurring in 55 (43.7%) supraorbital margins, followed by a notch with an accessory foramen in 20 (15.9%), a complete supraorbital foramen in 25 (19.8%), and co-occurrence of a notch with a complete foramen in 14 (11.1%). Incomplete supraorbital foramina accounted for 6 (4.8%), whereas supraorbital depression was observed in 4 (3.2%). Bony spurs associated with either the supraorbital foramen or notch were rare, occurring in 1 (0.8%) case each. 

**Table 1:** Distribution of supraorbital openings (N = 126)

Morphological Type	Right, n (%)	Left, n (%)	Total, n (%)
Supraorbital notch	25 (39.7%)	30 (47.6%)	55 (43.7%)
Co-occurrence of supraorbital notch with complete foramen	6 (9.5%)	8 (12.7%)	14 (11.1%)
Notch with accessory foramen	11 (17.5%)	9 (14.3%)	20 (15.9%)
Complete supraorbital foramen	15 (23.8%)	10 (15.9%)	25 (19.8%)
Incomplete supraorbital foramen	4 (6.3%)	2 (3.2%)	6 (4.8%)
Supraorbital foramen with bony spur	0 (0.0%)	1 (1.6%)	1 (0.8%)
Supraorbital notch with bony spur	0 (0.0%)	1 (1.6%)	1 (0.8%)
Supraorbital depression	2 (3.2%)	2 (3.2%)	4 (3.2%)
Total	63 (100.0%)	63 (100.0%)	126 (100.0%)

**Figure 1:**
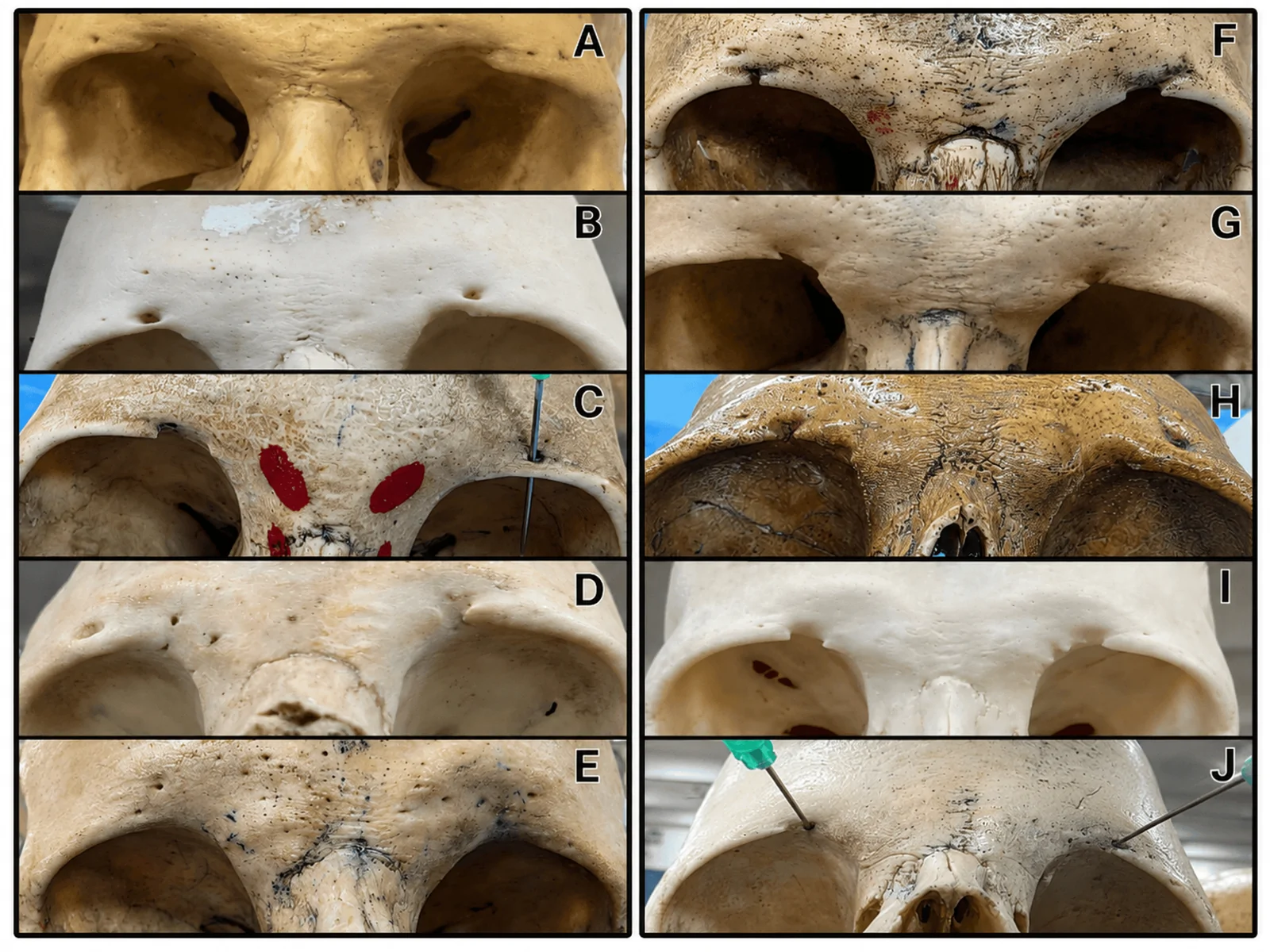
Morphological variations of the supraorbital openings (A) Bilateral supraorbital notches. (B) Bilateral supraorbital foramina. (C) Right supraorbital notch with left supraorbital foramen. (D) Right supraorbital foramen with left supraorbital notch. (E) Bilateral shallow supraorbital depressions without distinct notch or foramen. (F) Incomplete supraorbital foramen on the right side with a well-defined supraorbital notch on the left side. (G) Right-sided supraorbital notch with an incomplete supraorbital foramen on the left side. (H) Incomplete supraorbital foramen on the right side with coexistence of a notch and an accompanying complete foramen on the left side. (I) Right-sided supraorbital notch and left-sided supraorbital notch with a bony spur. (J) Bilateral supraorbital notches with accessory foramen.

Overall, notch-related variants constituted the predominant morphology of the supraorbital margin (Table 2).

**Table 2:** Overall distribution of supraorbital margin morphology (N = 126) Data are presented as frequencies (n) and percentages (%). Percentages are calculated based on the total number of supraorbital margins.

Morphological Category	Variants Included	Total, n (%)
Notch variants	Supraorbital notch, Notch with accessory foramen, Supraorbital notch with bony spur	76 (60.3%)
Foramen variants	Complete supraorbital foramen, incomplete supraorbital foramen, supraorbital foramen with bony spur	32 (25.4%)
Co-occurrence of notch–foramen variant	Combined supraorbital notch and complete supraorbital foramen	14 (11.1%)
Supraorbital depression	Smooth supraorbital margin with concavity	4 (3.2%)
Total	—	126 (100.0%)

Accessory supraorbital foramina

Accessory supraorbital foramina were identified in 20 (15.9%) of the 126 supraorbital margins (Table 1). A single accessory foramen was present in 14 (70.0%) supraorbital margins, whereas double accessory foramina were observed in 6 (30.0%) supraorbital margins. The lateral position was the most common, occurring in 10 (50.0%) supraorbital margins, followed by the superolateral position in 6 (30.0%) and medial position in 4 (20.0%) supraorbital margins (Table 3). 

**Table 3:** Distribution of accessory supraorbital foramina (N = 20) Data are presented as frequencies (n) and percentages (%). Percentages are calculated based on the total number of accessory foramina (N = 20).

Variable	Right, n (%)	Left, n (%)	Total, n (%)
Accessory foramina present	11 (55.0%)	9 (45.0%)	20 (100.0%)
Number			
Single	8 (40.0%)	6 (30.0%)	14 (70.0%)
Double	3 (15.0%)	3 (15.0%)	6 (30.0%)
Location			
Medial	2 (10.0%)	2 (10.0%)	4 (20.0%)
Lateral	6 (30.0%)	4 (20.0%)	10 (50.0%)
Superolateral	3 (15.0%)	3 (15.0%)	6 (30.0%)

Morphological configuration of complete supraorbital foramina

Among the 39 complete supraorbital foramina, the oval configuration was most common (19, 48.7%), followed by the round configuration (11, 28.2%) and the irregular configuration (9, 23.1%) (Table 4). 

**Table 4:** Morphological configuration of complete supraorbital foramina (N = 39) Data are presented as frequencies and percentages.

Shape	Right, n (%)	Left, n (%)	Total, n (%)
Oval	10 (25.6%)	9 (23.1%)	19 (48.7%)
Round	6 (15.4%)	5 (12.8%)	11 (28.2%)
Irregular	5 (12.8%)	4 (10.3%)	9 (23.1%)
Total	21 (53.8%)	18 (46.2%)	39 (100.0%)

Morphometric characteristics

Morphometric measurements of complete supraorbital foramina are summarized in Table 5.

**Table 5:** Morphometric measurements of the supraorbital foramen (N = 39) Values are expressed as mean ± standard deviation (SD) in millimeters (mm). Right–left differences were assessed using an independent-samples t-test. p < 0.05 was considered statistically significant.

Measurement	Right, Mean ± SD	Left, Mean ± SD	t	p
Vertical diameter	2.14 ± 0.58	2.27 ± 0.93	0.528	0.602
Transverse diameter	3.13 ± 0.91	2.94 ± 0.93	0.662	0.512
Vertical distance from inferior margin of SOF to superior orbital rim	3.13 ± 0.98	3.01 ± 1.27	0.328	0.744

The morphometric analysis showed that the mean vertical diameter was slightly greater on the left side (2.27 ± 0.93 mm) than on the right side (2.14 ± 0.58 mm); however, this difference was not statistically significant (p = 0.602). Similarly, the mean transverse diameter was slightly greater on the right side (3.13 ± 0.91 mm) compared with the left side (2.94 ± 0.93 mm), but the difference was also not statistically significant (p = 0.512). The vertical distance from the inferior margin of the supraorbital foramen to the superior orbital rim was 3.13 ± 0.98 mm on the right and 3.01 ± 1.27 mm on the left (p = 0.744). No statistically significant difference was detected between the right- and left-sided measurements of the vertical or transverse diameter of the supraorbital foramen.

Relationship to anatomical landmarks

Linear measurements are presented in Table 6. The paired-samples t-test demonstrated a statistically significant difference between the right- and left-sided distances from the nasion to the center of the supraorbital opening (SOF/SON/SOD) (t = 3.799, p < 0.001). In contrast, no statistically significant differences were observed for the distance from the center of the supraorbital opening (SOF/SON/SOD) to the frontozygomatic suture (t = 1.495, p = 0.140) or the distance from the center of the SOF/SON/SOD to the temporal crest of the frontal bone (t = 0.115, p = 0.909). These findings indicate that, among the three parameters assessed, only the distance from the nasion to the center of the supraorbital opening showed a significant right-left asymmetry.

**Table 6:** Linear measurements of the supraorbital foramen/notch/depression (SOF/SON/SOD) relative to anatomical landmarks (N = 126) Right–left differences were assessed using a paired-samples t-test. p-values are two-tailed, with p < 0.05 considered statistically significant.

Measurement	Right, Mean ± SD	Left, Mean ± SD	t	p
Distance from the nasion to the center of the supraorbital opening (SOF/SON/SOD)	22.63 ± 1.55	21.97 ± 1.87	3.799	<0.001
Distance from the center of the supraorbital opening (SOF/SON/SOD) to the frontozygomatic suture	28.43 ± 1.37	28.12 ± 1.43	1.495	0.14
Distance from the center of the SOF/SON/SOD to the temporal crest of the frontal bone	28.54 ± 3.52	28.48 ± 3.36	0.115	0.909

## Discussion

The supraorbital foramen/notch (SOF/SON) is an important anatomical landmark that transmits the supraorbital neurovascular bundle, which provides sensory innervation to the forehead and anterior scalp. Accurate knowledge of its morphology and location is essential for regional anesthesia, craniofacial surgery, orbital procedures, and radiological interpretation.

In the present study, notch variants constituted the predominant morphology, accounting for 76 (60.3%), followed by foramen variants at 32 (25.4%). Combined occurrence of a supraorbital notch and complete supraorbital foramen was observed in 14 (11.1%) skulls, while supraorbital depression without a distinct notch or foramen accounted for 4 (3.2%). Embryologically, the supraorbital foramen develops through ossification of the periosteal ligament bridging the supraorbital notch. Complete ossification results in formation of a foramen, whereas incomplete ossification leads to persistence of the notch, thereby contributing to population-specific morphological variation [4].

The predominance of notch variants observed in the present North Indian population is comparable to previous Indian studies by Sharma et al. (70.5% notches and 29.5% foramina) [20] and Kumar et al. (63.2% notches) [17]. However, the frequency of notches was considerably higher than that reported by Jaiswal et al. (43.8%) [21] and Singh (41.95%) [11]. Similar findings have been reported from neighboring South Asian populations, including Sri Lankan skulls studied by Nanayakkara et al., who observed 73.8% notches and 26.2% foramina [1]. Comparable distributions have also been described in Thai (66.5% notches) [18] and Bosnian (73.8% notches) [4] populations. In contrast, Korean populations demonstrate a substantially higher prevalence of complete supraorbital foramina, with single foramina occurring in approximately 39.7% and, in some studies, exceeding the frequency of notches [22].

The predominance of notch variants in the present study is consistent with observations from populations inhabiting relatively warmer climatic regions. Tomaszewska et al. proposed that complete foramina may provide greater protection to the supraorbital neurovascular bundle in colder environments, whereas notches may facilitate heat dissipation in warmer climates [23]. However, the present study was not designed to investigate climatic adaptation directly, and therefore this hypothesis should be interpreted cautiously.

Morphometric evaluation demonstrated that the mean distance from the nasion to the center of the supraorbital opening was 22.63 ± 1.55 mm on the right side and 21.97 ± 1.87 mm on the left side. These findings are comparable with previous Indian studies, including those by Jaiswal et al. (21.6 mm) [21] and Sharma et al. (20.11-21.94 mm) [20]. However, the supraorbital opening was positioned more medially than reported in Thai (25.14 mm) [18] and Korean (27.19 mm) [22] populations. A statistically significant side-to-side difference was observed (p < 0.001), with the right supraorbital opening located slightly more laterally than the left. A similar asymmetry has also been reported by Sharma et al. [20].

Although the nasion is commonly used as a reference point for morphometric studies, it may be difficult to identify accurately during surgical procedures. More reliable intraoperative landmarks include the frontozygomatic suture (FZS) and the temporal crest of the frontal bone (TCFB), both of which are readily palpable [24]. In the present study, the mean distance from the center of the supraorbital opening to the frontozygomatic suture was 28.43 ± 1.37 mm on the right side and 28.12 ± 1.43 mm on the left side. The corresponding distance to the temporal crest of the frontal bone was 28.54 ± 3.52 mm on the right side and 28.48 ± 3.36 mm on the left side. No significant bilateral differences were observed, suggesting that these landmarks may provide consistent reference points during bilateral craniofacial procedures, including brow lift surgery and frontal craniotomy.

The mean vertical distance from the inferior margin of the supraorbital opening to the superior orbital rim was 3.13 ± 0.98 mm on the right side and 3.01 ± 1.27 mm on the left side. The right side therefore showed a slightly higher mean value than the left side; however, the difference was not statistically significant (p = 0.744). These findings closely resemble those reported in Thai skulls (3.15 mm) [18] but are slightly greater than measurements reported in Sri Lankan [1] and Caucasian populations [2]. Clinically, when the supraorbital neurovascular bundle exits through a complete foramen, its mobility is relatively restricted compared with a notch. Consequently, surgical dissection performed within approximately 3 mm superior to the orbital rim requires particular caution to minimize the risk of nerve traction, compression, or transection, thereby reducing the likelihood of postoperative sensory deficits or neuralgia.

Accessory supraorbital foramina were identified in 20 (15.9%) of 126 supraorbital regions. This prevalence is comparable with reports from Bosnian (16.67%) [4] and Sri Lankan (18.9%) [1] populations but exceeds that reported in Thai populations (8.0%) [18]. Most accessory foramina were located lateral to the principal supraorbital opening, occurring in 10 (50.0%), followed by the superolateral position in 6 (30.0%) and medial position in 4 (20.0%) [1]. From a clinical perspective, accessory foramina may transmit additional branches of the supraorbital nerve, potentially resulting in incomplete anesthesia following conventional supraorbital nerve block techniques. Furthermore, these supernumerary openings should be recognized during reconstructive, orbital, and cosmetic procedures to minimize inadvertent neurovascular injury.

The present findings also provide valuable population-specific morphometric data that may contribute to comparative anatomical, anthropological, and forensic investigations. Nevertheless, supraorbital morphology demonstrates considerable overlap among populations and should not be considered an independent marker for ancestry estimation or forensic identification.

Limitations

This study was based on dry skulls from a single institutional collection, limiting generalizability. Sex and age data were unavailable, and no radiological or cadaveric correlation was performed. The relatively modest sample size also limited analysis of less frequent variants.

## Conclusions

The present study provides descriptive morphological and morphometric data on the supraorbital foramen and notch in an osteological sample from Uttar Pradesh and adjacent regions. Supraorbital notch variants predominated, accessory supraorbital foramina were identified in a proportion of examined supraorbital sides, and measurable bilateral variation was observed in the relationship of the supraorbital opening to anatomical landmarks. These findings provide anatomical data that may be useful for comparative and clinical anatomical research; however, their applicability to broader North Indian populations and their direct clinical or forensic utility cannot be established from the present study alone. The single-institution skeletal collection, modest sample size, lack of individual chronological age data, absence of sex-stratified analysis, and lack of radiological or clinical correlation limit generalizability. Larger, geographically diverse, and demographically characterized skeletal studies, preferably incorporating radiological and clinical validation, are required to establish more broadly representative reference data and determine their clinical applicability.
